# Design and characterisation of a dissolving microneedle patch for intradermal vaccination with heat-inactivated bacteria: A proof of concept study

**DOI:** 10.1016/j.ijpharm.2018.07.049

**Published:** 2018-10-05

**Authors:** Aoife M. Rodgers, Maelíosa T.C. McCrudden, Eva.M. Vincente-Perez, Alice V. Dubois, Rebecca J. Ingram, Eneko Larrañeta, Adrien Kissenpfennig, Ryan F. Donnelly

**Affiliations:** a School of Pharmacy, Medical Biology Centre, Queens University Belfast, 97 Lisburn Road, Belfast BT9 7BL, United Kingdom; b Centre for Experimental Medicine, School of Medicine, Dentistry & Biomedical Science, Queens University Belfast, BT9 7BL, United Kingdom

**Keywords:** Vaccine, Skin, Heat-inactivated bacteria, Intradermal, Microneedle

## Abstract

This work describes the formulation and evaluation of dissolving microneedle patches (MNs) for intradermal delivery of heat-inactivated bacteria. *Pseudomonas aeruginosa,* strain PA01, was used as a model bacterium. Utilising a simple, cost effective fabrication process, *P. aeruginosa* was heat-inactivated and formulated into dissolving MNs, fabricated from aqueous blends of 20% w/w poly(methylvinylether/maleic acid). The resultant MNs were of sufficient mechanical strength to consistently penetrate a validated skin model Parafilm M®, inserting to a depth of between 254 and 381 µm. MNs were successfully inserted into murine skin and partially dissolved. Analysis of MN dissolution kinetics in murine ears *via* optical coherence tomography showed almost complete MN dissolution 5 min post-insertion. Mice were vaccinated using these optimised MNs by application of one MN to the dorsal surface of each ear (5 min). Mice were subsequently challenged intranasally (24 h) with a live culture of *P. aeruginosa* (2 × 10^6^ colony forming units). Bacterial load in the lungs of mice vaccinated with *P. aeruginosa* MNs was significantly (*p =* 0*.*0059) lower than those of their unvaccinated counterparts. This proof of concept work demonstrates the potential of dissolving MNs for intradermal vaccination with heat-inactivated bacteria. MNs may be a cost effective, potentially viable delivery system, which could easily be implemented in developing countries, allowing a rapid and simplified approach to vaccinating against a specific pathogen.

## Graphical abstract

**Figure:**
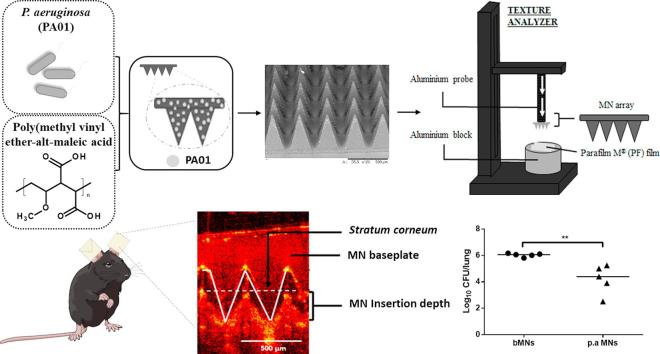

## 1 Introduction

Vaccination is undoubtedly one of the greatest breakthroughs of modern medicine. Despite this, there remain significant challenges surrounding vaccine distribution, delivery and efficacy, particularly within developing countries (Oyston and Robinson, 2012). Most vaccines are administered *via* a hypodermic needle, necessitating trained personnel for vaccine reconstitution and administration. The hypodermic needle also results in the creation of biohazardous sharps waste, which pose the risk of infection to both patients and healthcare workers. An estimated 33,800 HIV infections, 1.7 million hepatitis B infections and 315,000 hepatitis C infections annually are caused from unsafe injection practices (WHO | Injection safety policy and global campaign,” 2016). Moreover, needle phobia often results in poor patient compliance (Taddio et al., 2012).

To retain their potency, many vaccines require cold chain storage, from their distribution through to their administration. This has associated cost challenges to low-resource countries and presents other challenges in relation to geographical accessibility. The estimated cost of cold chain storage is $200–$300 million annually and cold chain failures can result in vaccine shortages (Burstein et al., 2013). In addition, many vaccine formulations necessitate booster injections in order to mount an appropriate immune response, which can be difficult to implement, subsequently resulting in poor vaccine coverage. The World Health Organisation (WHO) estimates that the total number of children who die annually from vaccine-preventable diseases is 1.5 million, accounting for 17% of all under 5 deaths (“Global Immunization Data,” 2014). Thus, there is a pressing need to develop safe, cost-effective, innovative vaccine formulation strategies, to improve coverage of currently available vaccines, while also developing vaccines for infections for which no currently available vaccine exists, to help control the incidence of infectious diseases.

The skin represents an attractive route for vaccination, having the advantage of increased patient compliance and increased vaccine effectiveness. The skin is densely populated with professional antigen presenting cells (APCs). Dendritic cells (DCs) present in the dermis and Langerhans cells (LCs) in the epidermis play a pivotal role in induction of immune responses, whereby they capture foreign antigens and present them in draining lymph nodes (Zaric et al., 2013a). The skin, by virtue of its function in protection against water loss and environmental influences, presents a major obstacle for intradermal (i.d) delivery (Elias, 2008, Prausnitz et al., 2012). Human skin is composed of two main compartments namely, the epidermis and the dermis. The skin’s barrier function is almost entirely attributable to the outermost layer of the epidermis, the *stratum corneum* (*SC*).

Dissolving microneedle patches (MNs) have been proposed as a novel approach to increase skin permeability and may offer an ideal platform for i.d delivery (Ding et al., 2009, Rouphael et al., 2017). MNs are minimally invasive devices that consist of a series of microscopic projections attached to a base support (McCrudden et al., 2014, McCrudden et al., 2014b). Upon application to the skin surface, MNs create microscopic holes in the skin, bypassing the *SC,* and subsequently delivering their payload into viable skin (Zaric et al., 2013b). MNs are typically fabricated such that they are long enough to bypass the *SC,* yet are short enough to avoid stimulation of dermal nerves. As such, they provide a painless, minimally invasive method of delivery that is well accepted by human subjects (Arya et al., 2017, Ripolin et al., 2017). MNs are advantageous for vaccination; due to direct targeting of skin APC, they are dose sparing, and offer improved protection, while concurrently reducing the requirement for usage by trained personnel (Quan et al., 2010, Ripolin et al., 2017, Zaric et al., 2015). Further to this, MNs provide a more simplified supply chain due to their ease of storage, distribution and disposal due to avoidance generation of sharps wastes (Arya and Prausnitz, 2016).

Dissolving MNs have received substantial interest for delivery of currently licenced vaccines. For example, Matsuo et al. demonstrated vaccine efficacy against tetanus and diphtheria, malaria and influenza using dissolving MNs (Matsuo et al., 2012). Moreover, a more recent study by Rouphael and co-workers showed that dissolving MNs for influenza vaccination was well tolerated and generated robust antibody responses in humans (Rouphael et al., 2017). However, there has been less research into the use of MNs for development of novel vaccines. The objective of the present study was to design and evaluate a dissolving MN laden with heat-inactivated bacteria, as a novel, simplified approach to vaccinating against a specific bacterium. *Pseudomonas aeruginosa* strain PA01, was chosen as a model bacterium. Utilising a simple, cost effective fabrication process, *P. aeruginosa* was heat-inactivated and formulated into dissolving MNs. As MNs offer the possibility for self-application, the expected benefit of MN-mediated vaccination with heat-inactivated bacteria would lie in the simplicity and cost effectiveness from the practical perspective. Whole heat-inactivated vaccines are stable, safe for administration to immunocompromised patients and can be easily freeze dried and incorporated into MNs, thus allowing ease of transport and distribution, and a cost-effective approach to vaccinating against a specific pathogen. This approach could potentially allow for mass immunisation within developing countries, helping increase vaccine coverage. To the best of our knowledge, this is the first study that uses dissolving MN arrays for delivery of a heat-inactivated bacterium, intended for vaccination against a bacteria for which no currently licenced vaccine exists.

## 2 Materials and methods

### 2.1 Materials

Gantrez® S-97 (PMVE/MA), a copolymer of methyl-vinyl-ether and maleic acid (MW 1,500,000 Da), was provided by Ashland (Tadworth, Surrey, UK). Parafilm M®, 127 µm thick, was obtained from BRAND GMBH (Wertheim, Germany). Dulbecco’s phosphate buffered saline (PBS) (endotoxin tested), Luria-Bertani broth and cetrimide agar were obtained from Sigma-Aldrich (Dorset, UK). All other chemicals were of analytical reagent grade. Rompun™ 100 mg/ml, was purchased from Bayer Healthcare LLC (Bergkamen Germany). Ketalar™ 10 mg/ml ketamine hydrochloric solution was purchased from Pfizer Ltd (Ramsgate Road, Kent, UK).

### 2.2 Preparation of *P. aeruginosa*: culture, heat-inactivation and lyophilisation

*P. aeruginosa* (strain PAO1), was grown on cetrimide agar at 37 °C overnight. Post incubation a colony was selected and cultured overnight in Luria-Bertani (LB) broth (5 ml) at 37 °C in a rotatory incubator shaker (180 rpm). Post incubation, the culture (5 ml) was transferred into an Erlenmeyer flask containing a further 995 ml of LB broth and incubated as above. After incubation, the culture was transferred to 50 ml Falcon tubes and the bacteria was collected by centrifugation (4000 rpm for 20 min), and the supernatant was subsequently removed. The resultant *P. aeruginosa* pellets were washed twice by addition of phosphate buffer (PB), followed by centrifugation as described above. Post washing, the pellets were re-suspended in PB buffer and heat-killed by submersion in a water-bath (65 °C) for 1 h. The suspension was inoculated onto LB agar to ensure bacteria were not viable. The remaining suspension was placed in a freezer (−80 °C) for 1 h. Once frozen, each Falcon tube lid was replaced with a KimWipe (Kimberly-Clark Co., USA) and the tubes were transferred to a side arm of a manifold-type freeze dryer (MiVac freeze dryer) under a vacuum for 48 h before being removed and stored until further use.

### 2.3 Formulation and fabrication of polymeric MN arrays

**Fig. 1:**
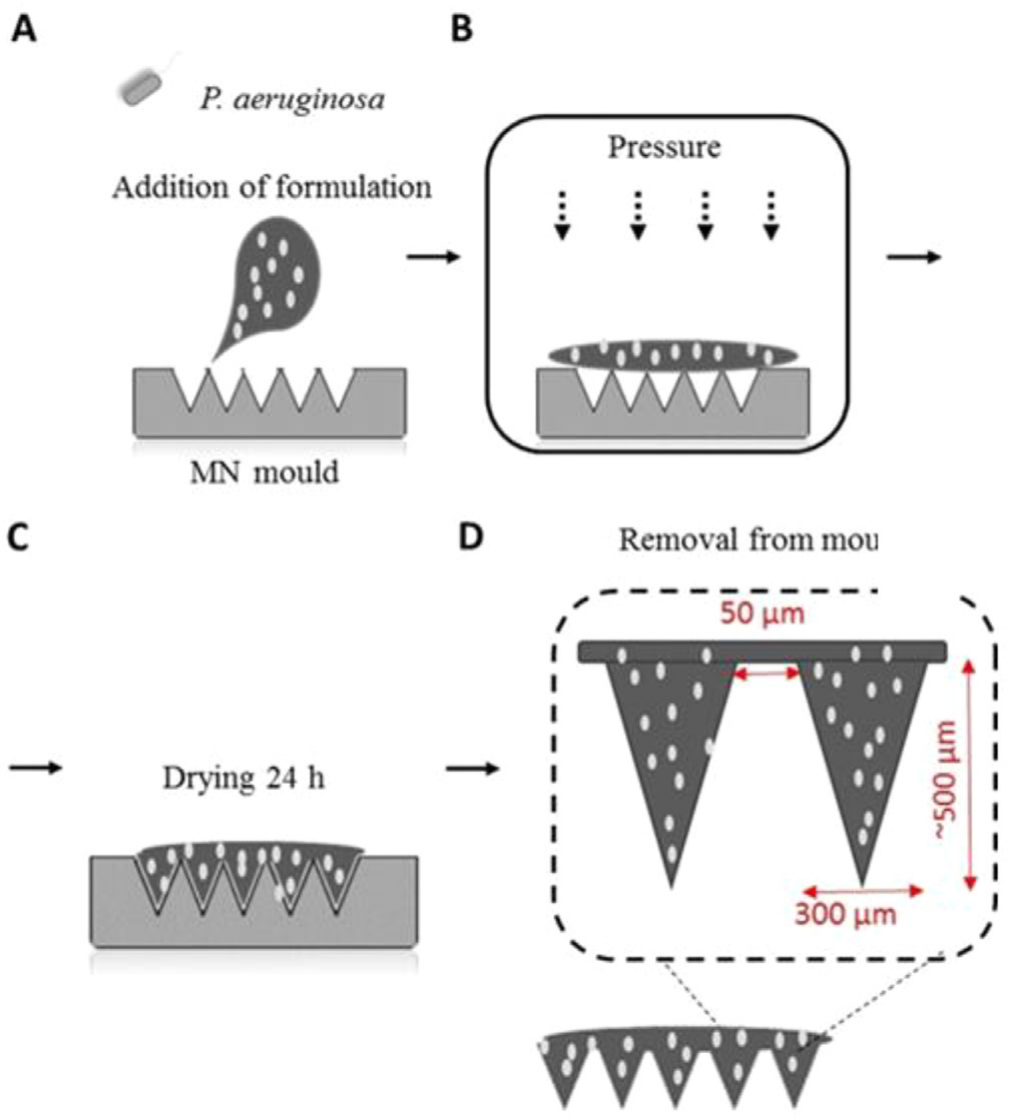
A schematic representation of the steps involved in fabrication of dissolving MN laden with heat-inactivated *Pseudomonas aeruginosa*.

MNs were prepared from aqueous blends of Gantrez® S-97. A stock of Gantrez® S-97 (40%) was diluted with the appropriate volume of a suspension of *P. aeruginosa* diluted in phosphate buffered saline (PBS), pH 7.4, to give a final concentration of 20% Gantrez®S-97. One hundred milligrams of this suspension, containing 200 µg of heat-inactivated *P. aeruginosa* was poured onto silicon micromoulds. The micromoulds contained 361 (19 × 19) pyramidal shaped needles, perpendicular to the base, 500 µm in height, 300 µm wide and with 50 µm interspacing. The arrays had areas of 0.6 cm^2^. A pressure of 3 bar was subsequently used to force the MN formulation into the needles of the MN mould. The MNs were dried under controlled temperature conditions (19 °C) for 24 h, before being carefully removed from the MN moulds and stored until further use. A schematic representation showing the method of production of dissolving MNs is shown in Fig. 1**.**

### 2.4 Scanning electron microscopy for imaging of MN arrays

When imaging MNs using scanning electron microscopy (SEM), MNs were mounted onto an aluminium stub and observed under a TM3030 microscope (Hitachi Ltd, Japan). Micrographs of MN structures were obtained and the surface morphology and height/width of individual needles were examined/measured.

### 2.5 Mechanical characterisation of MNs

MNs were subjected to mechanical testing, as previously described (McCrudden et al., 2014, McCrudden et al., 2014b). Mechanical testing of the MNs was performed using a TA.XTPlus Texture Analyser (Stable MicroSystems, Surrey, UK) in compression mode. MNs were attached to the cylindrical probe of the TA. The probe was lowered vertically at a speed of approximately 1.19 mm s^−1^ until the required force of 32 N/array was applied. This has been shown to be the average maximum force a human exerts when applying MNs (Larrañeta et al., 2014). Force was applied to the MNs for 30 s, the time recommended for MN application (Donnelly et al., 2014), and the probe was subsequently retracted from the testing area. MNs were visually examined pre- and post-application of the compression load using a light microscope (GXMGE-5 digital microscope, Laboratory Analysis Ltd, Devon, UK). The resulting images were used to measure the heights of individual needles using ImageJ® (National Institute of Health, Bethesda, USA) software. The percentage change in MN height was subsequently calculated using Eq. (1) below, where H_BC_ is the needle heights before compression and H_AC_ is the needle heights after compression.

(1) $$\%\text{Compression}\;=\frac{\text{HBC}-\text{HAC}}{\text{HBC}}\times 100$$

MN insertion testing was carried out as previously described by Larreneta et al*.* whereby Parafilm M® (PF), a blend of a hydrocarbon wax and a polyolefin, was used as a skin simulant (Larrañeta et al., 2014). For this purpose, 8 PF layers were combined to form a PF film of approximately 1 mm thickness. Prior to insertion, MNs were analysed using a Leica EZ4 D digital microscope (Leica, Wetzler, Germany). The Texture Analyser (TA) insertion was then performed in compression mode. Briefly, MNs were attached to the cylindrical probe of the TA and a force was applied to the MNs. MN arrays were subsequently removed from the PF sheet and each PF layer was unfolded. The holes created in each layer were imaged using a Leica EZ4 D digital microscope (Leica, Wetzler, Germany). For detection of the MN insertion holes, MNs were placed between two polarized filters. The number of holes created in each layer as a measure of insertion depth was subsequently calculated.

### 2.6 Analysis of MN dissolution using optical coherence tomography

The *in vivo* dissolution rate of MN inserted into murine ears was conducted to determine the optimal MN application time for subsequent *in vivo* studies. Briefly, MN arrays were attached to translucent double-sided tape and firmly attached to the dorsal surface of each murine ear. MNs were inserted by application of thumb pressure to the MN array (30 s). Inserted MNs were immediately viewed using an EX1301 VivoSight® OCT Microscope with a hand-held probe, the contacting surface of which had an area of 1 cm^2^. The swept-source Fourier domain OCT system has a laser centre wave-length of 1305 ± 15 nm, facilitating real-time high resolutions imaging of the upper skin layers (<7.5 µm lateral and <10 µm vertical resolution). The resulting 2D images were analysed using ImageJ® (National Institute of Health, Bethesda, USA) software. Obtained image files had a scale of 1.0 pixel = 4.2 µm, allowing accurate measurements of MN insertion depth. Five replicates were performed per MN formulation and for each MN inserted, 20 measurements of individual MN insertion depths were taken by random selection of needles, from the 361 penetrating MNs in each array. MN insertion data was presented as (±SD) of 20 replicate measurements. The procedure was repeated at pre-determined time points (5, 15, 30, 45, 60 min).

### 2.7 MN-mediated vaccination studies

*In vivo* studies were performed on C57/BL6 mice, 6–8 weeks old. Mice were randomly divided into 2 groups, those that received blank MNs (bMNs), or *P. aeruginosa* MNs (p.a MNs), n = 5/group. Briefly, mice were anaesthetised intraperitoneally (i.p) and immunised by manual application of one MN to the dorsal surface of each ear for 5 min, resulting in a corresponding dose of 400 µg of heat killed *P. aeruginosa****.*** At 7 days post MN application, both vaccinated and un-vaccinated mice were challenged with 2 × 10^6^ colony forming units (CFU) of *P. aeruginosa* (PA01), delivered intranasally (i.n). Each mouse received a 20 µl volume of bacterial suspension, diluted in endotoxin-free PBS and administered with a micropipette. Mice were carefully monitored over the course of infection and weights were assessed. Subsequently, 24 h post-challenge, mice were sacrificed and the lungs and spleens were harvested for analysis of CFU counts.

#### 2.7.1 Culture of *P. aeruginosa* for challenge study

*P. aeruginosa* was grown on cetrimide agar at 37 °C overnight. Preparation of bacterial inoculum for challenge study was performed by transferring a colony of *P. aeruginosa* into LB broth. The culture was grown overnight at 37° C under gentle agitation. Following overnight incubation, the bacteria were harvested by centrifuged for 25 min at 22° C 4000 rpm and the supernatant was carefully removed. The pellet was subsequently washed in PBS. Finally, optical density (OD) measurements were used to dilute the bacterial suspension to the desired concentration (10^6^ CFU) using PBS and this was confirmed by plating serial dilutions of the preparations for enumeration of CFU post-overnight incubation.

#### 2.7.2 Analysis of colony forming units in the lungs and spleens 24 h post bacterial inoculation

Lung and spleen tissue was homogenized under aseptic conditions in 1 ml of PBS in a sterile bijou using a mechanical homogeniser. Serial dilutions of the homogenized organs (100 µl) were plated onto cetrimide agar (Sigma-Aldrich Dorset, UK) for analysis of bacterial load*.* The agar plates were incubated overnight at 37 °C and the number of CFUs counted for determination of bacterial load.

### 2.8 Statistical analysis

Data was compared using an unpaired, two-tailed student’s *t*-test. In all cases, *p* < 0.05 was considered acceptable for rejection of the null hypothesis. Statistical analysis was carried out using GraphPad Prism version 5.0 (GraphPad Sotware Inc., San Diego, California).

## 3 Results

### 3.1 MN formulation and fabrication

**Fig. 2:**
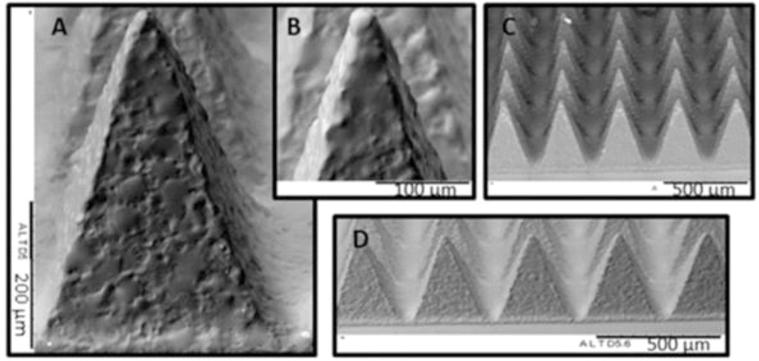
Representative SEM images of MNs prepared from PMVE/MA laden with P. aeruginosa, showing: (A) fully formed individual needle, approximately 500 µm in height and a width at base of 300 µm, (B) needle tip, (C) fully formed MN array and (D) row of individual needles. Scale bar as per images.

Fig. 2 shows representative SEM images of dissolving MNs prepared from PMVE/MA and laden with heat-inactivated *P. aeruginosa* (p.a MNs). The resulting MNs were approximately 500 µm in height, with a width at base of approximately 300 µm, and the fabrication process resulted in MNs with sharp needle tips, essential for insertion into murine skin and successful vaccine delivery. A complete array of needles was formed and MN baseplates were of sufficient strength to be easily removed from the moulds without damage being caused to the array. The addition of *P. aeruginosa* did not appear to affect MN structure, or the morphology of the individual needles.

### 3.2 Mechanical characterisation of MN arrays

**Fig. 3:**
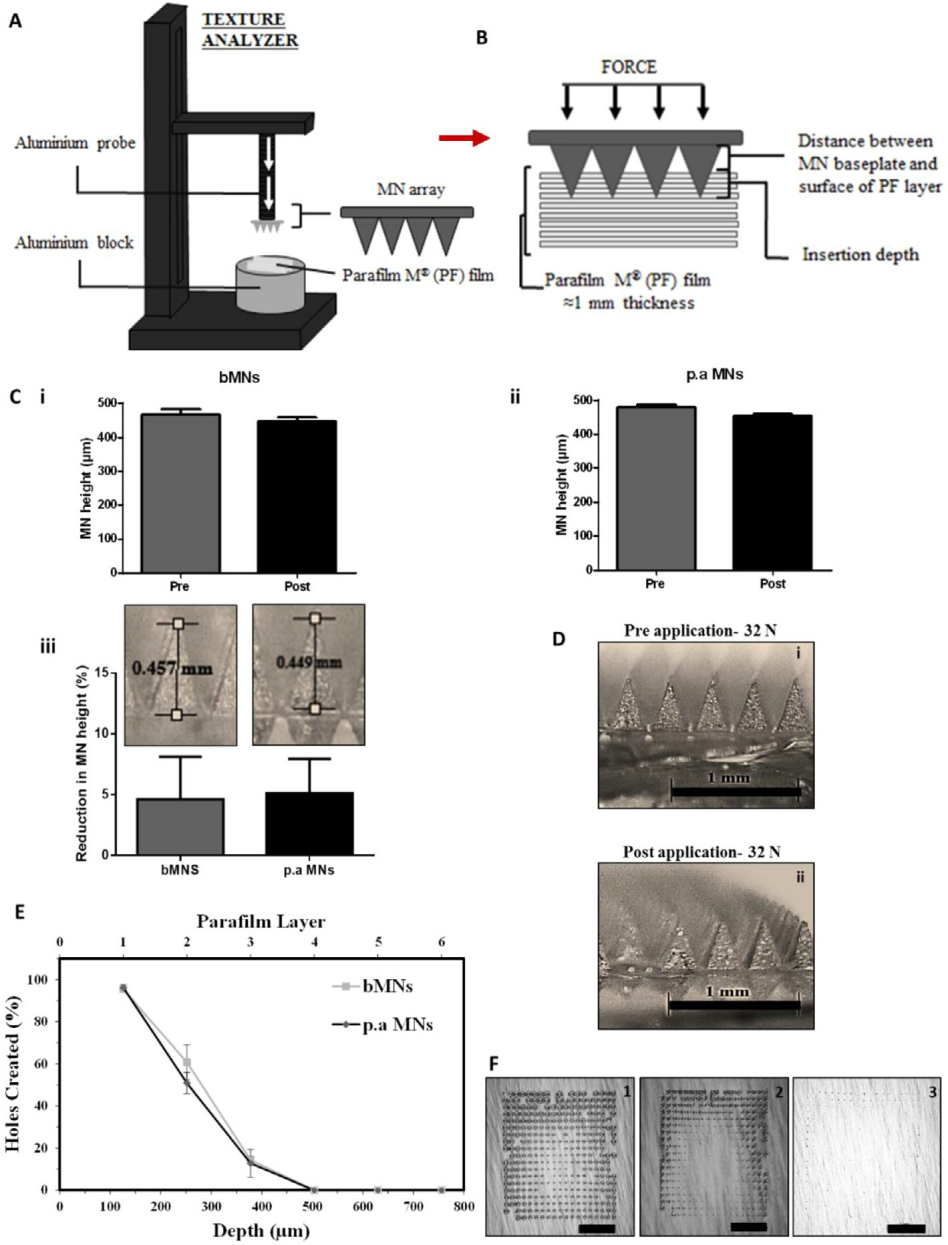
Mechanical characterisation of MN arrays. Diagrammatic representation of the MN compression (A) and insertion into Parafilm M® (B) test method. (C) MN height pre- and post-compression (32 N) at 1.19 m/s, held for 30 s (mean ± SD, n = 5), bMNs (i) and p.a MNs (ii). Comparison of percentage reduction in MN height of MN arrays prepared from different formulations (iii). (D) Representative images of p.a MNs pre- (i) and (ii) post-application of 32 N (the scale bars represent 1 mm). (E) Percentage of holes created in each Parafilm M® layer and their corresponding insertion depth, using a force of 32 N for bMNs and p.a MNs, mean ± SD, n = 5. (F) Representative microscope images of Parafilm M® layers after insertion of p.a MNs showing first (1), second (2), and third (3) Parafilm M® layers (the scale bar represents 2 mm).

Following application of the axial load, both MN types reduced in height. The blank MNs (bMNs) reduced in height by 4.4% (Fig. 3C-i) and the *P. aeruginosa* MNs (p.a MNs) by 4.7% (Fig. 3C-ii), respectively. Thus, there were no significant differences in the percentage reduction in MN height between the two MN formulations (Fig. 3C-iii) (*p =* 0.805)**,** indicating that the loading of *P. aeruginosa* within the MNs did not affect their rigidity or compression characteristics. None of the MNs, or the baseplates of the MNs fractured post-application of 32 N, nor did the MNs appear to be compressed (Fig. 3D).

Post-application of an insertion force of 32 N, both MN types inserted into the PF layers (Fig. 3E) and the percentage of holes created in each layer was subsequently analysed (Fig. 3F). Both MNs had similar insertion profiles inserting into two PF layers (∼55% needles inserted), with <20% inserting into the third layer. As such, it can be estimated that the MNs insertion depth was between 254 µm and 381 µm of the total 500-µm needle height. These results are concurrent with previously reported studies with dissolving MN insertion into PF, whereby the insertion was approximately 60% of the total needle height (González-Vázquez et al., 2017, Larrañeta et al., 2014).

Notably, upon removal of MNs from the PF layers, all needles remained attached to the baseplate and were not retained within the PF layers. It was concluded that the p.a MNs produced, had sufficient mechanical strength and insertion properties to successfully and consistently penetrate the skin simulant PF and in a manner similar to that of bMNs. From these results, it was deduced that the MNs produced should be of sufficient strength to penetrate the *SC in vivo.* Furthermore, the insertion and compression results obtained herein are comparable to those of dissolving MNs previously used by our Group for successful *in vitro* and *in vivo* delivery of various compounds, including antibiotics, such as gentamicin, cardiovascular drugs, and various model protein antigens (González-Vázquez et al., 2017; McCrudden et al., 2015; Quinn et al., 2015, Zaric et al., 2013a).

### 3.3 Microneedle in-skin dissolution studies

**Fig. 4:**
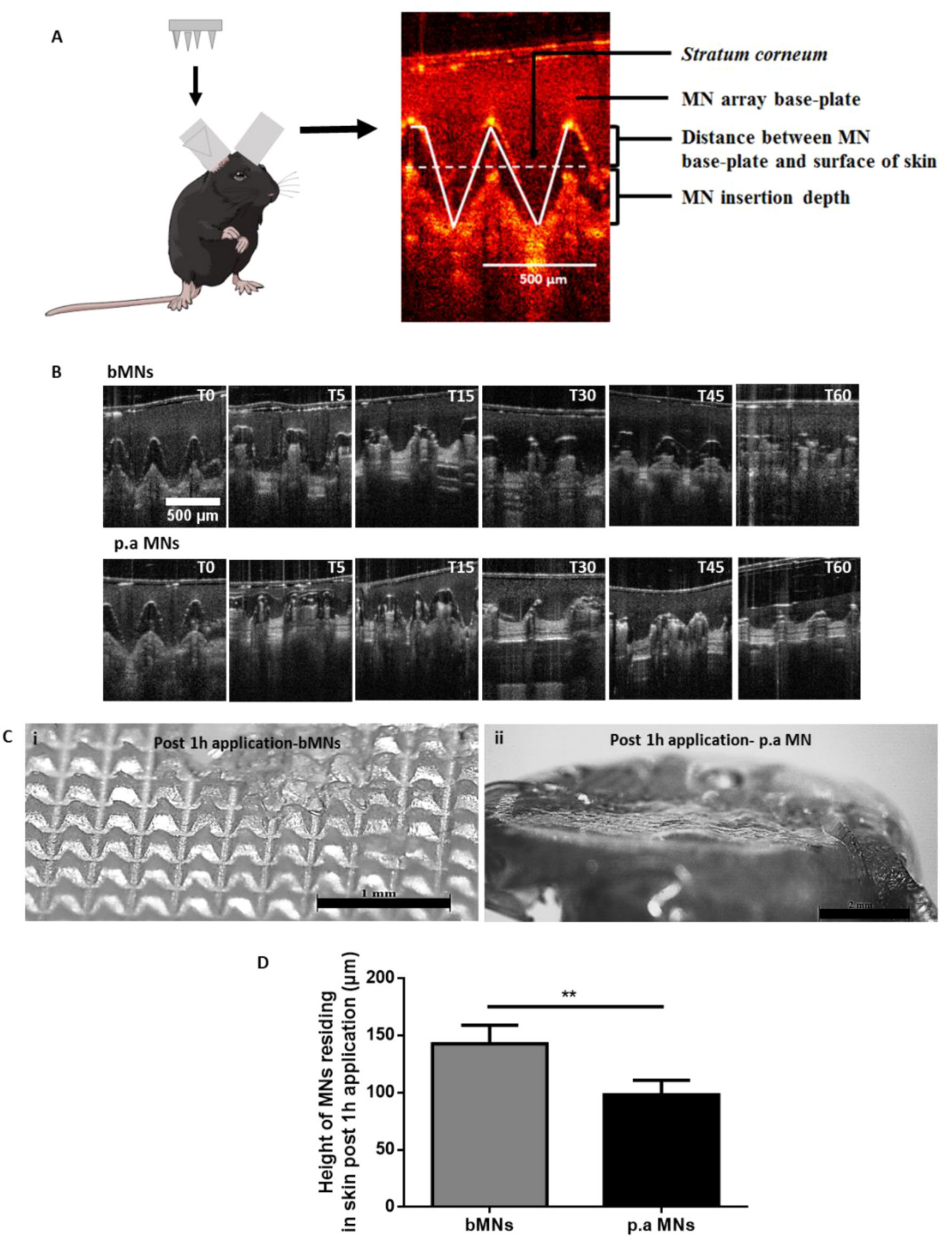
MN arrays efficiently insert and dissolve in murine skin. MNs were applied to the dorsal surface of murine ears as depicted in the schematic image, and OCT was subsequently used to image MNs inserted in the ears (A). Representative images of bMNs and p.a MNs inserted in skin showing MN dissolution as a function of time, (T = time, 0, 5, 15, 30, 45 and 60 min following skin insertion). (B), MNs were inserted in the skin for 1 h before being removed and imaged using a light microscope, (i) bMNs (ii) p.a MNs. Representative images of entire MN arrays post application in murine skin are shown in (the scale bars represent 1 mm in bMN image and 2 mm in p.a MN image) (C). Graph (D) shows the height of MNs residing in skin 1 h post application. Results are presented as mean ± SD, n = 5 MNs/group. For each MN array at least 20 individual needles were measured.

Upon application, both MN formulations successfully traversed the *SC* barrier and the needles penetrated into the skin layers*,* as highlighted in Fig. 4A. Furthermore, we evaluated the *in situ* dissolution rate of both bMNs and p.a MNs in murine ears. Dissolution of the MNs began immediately upon insertion into the skin and a gradual reduction in the height of the MNs penetrating the skin layers was observed over time (Fig. 4B). The p.a MNs had almost completely lost their needle profile and efficiently dissolved within the skin layers 5 min post-insertion (Fig. 4B). These results are in line with our previous study of dissolving MNs dissolution in murine skin (Zaric et al., 2013b). In contrast, the bMNs appeared to display slower dissolution kinetics and, after insertion into the skin for 15 min, the needles were still visible and retained their needle profile, thus suggesting that incorporation of the heat inactivated *P. aeruginosa* positively affected MN dissolution kinetics.

The OCT images showed no significant difference between the insertion profile of the bMNs and the p.a MNs. These results are concurrent with the MN characterisation results, whereby no difference between the mechanical characteristics, or insertion properties of the two MN formulations was observed in PF layers (Fig. 3). Moreover, examination of the height of MNs residing in skin 1 h post insertion was carried out to confirm the differences in dissolution kinetics between the two MNs formulations. In parallel to the OCT images, showing increased dissolution of the p.a MNs 1 h post insertion, the height of bMNs residing in skin was approximately 150 µm, whereas the height of the p.a MNs were all found to be <100 µm (Fig. 4D). Differences in MN dissolution were also evidenced by the microscopic images of both MN formulations 1 h post insertion in murine skin. Fig. 4C shows representative light microscope images of such MNs. These results imply that incorporation of *P. aeruginosa* into the MNs resulted in an increased MN dissolution rate compared to bMNs. Overall, the data presented here confirms that the polymeric MNs could successfully breach the *SC* barrier of murine ears consistently, dissolve within the skin layers releasing their payload and thus, could potentially be successfully used for i.d delivery of heat-inactivated bacteria.

### 3.4 MN-mediated vaccination studies: Investigating the protective efficacy of MN-mediated delivery of heat-inactivated *P. aeruginosa*

**Fig. 5:**
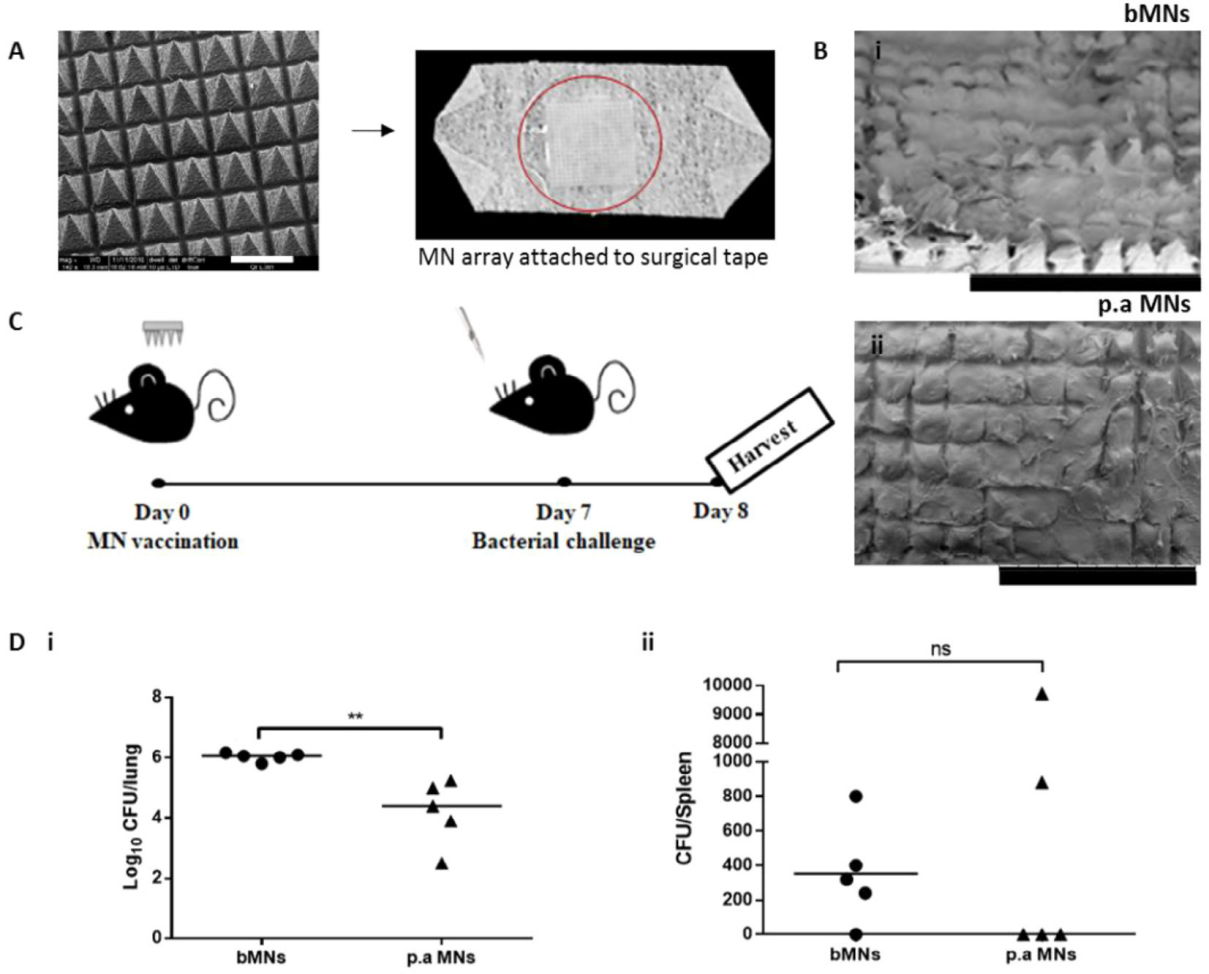
MN-mediated vaccination studies. MNs were attached to surgical tape (the scale bar represents 500 μm) (A) and applied to murine ears for 5 min before being removed and imaged via SEM to ensure complete dissolution (the scale bars represent 2 mm) (B). Post vaccination (day 7), mice were challenged for 24 h with a live culture of P. aeruginosa (PA01), delivered intra-nasally (106 CFU, 20 µl) (C). The lungs and spleens were harvested for analysis of CFUs (D). Bar represents geometric mean, n = 5 mice/group.

The most appropriate site for application of dissolving MNs for vaccine delivery was determined to be the dorsal surface of murine ears, an attractive tissue target due to the enriched network of resident APCs (Demuth et al., 2012, Zaric et al., 2013a). The MN application time determined to be appropriate was 5 min, as the greatest proportion of the length of each needle was dissolved within this time in the *in vivo* dissolution studies (Fig. 4). The attachment of MNs onto surgical tape and positioning of the MNs onto the dorsal surface of murine ears is exemplified in Fig. 5A. The attachment of MNs to surgical tape was required to hold the MNs firmly in place (5 min) for the vaccination procedure and one MN was applied to the dorsal surface of either mouse ear, before subsequently being removed and imaged to confirm dissolution and thus, successful delivery of heat inactivated *P. aeruginosa* (Fig. 5B). The total *P. aeruginosa* content loaded in each entire dissolving MN was approximately 200 µg. Therefore, as two dissolving MNs were applied, each mouse had approximately 400 µg heat-inactivated *P. aeruginosa* applied during the vaccination procedure.

Post-vaccination (7 days), mice underwent bacterial challenge by i.n inoculation with a live culture of *P. aeruginosa.* Bacterial clearance has been utilised as a measure of protection in vaccination trials and in *in vivo* challenge models (Cripps et al., 1994) and as such, was used as a measure of MN vaccination effectiveness. The efficacy of the polymeric MNs laden with heat-inactivated *P. aeruginosa* to induce enhanced bacterial clearance in the lungs of mice and to prohibit bacterial dissemination in the *P. aeruginosa* i.n challenge model was therefore evaluated. Mice were challenged for 24 h, after which lungs and spleens were harvested for analysis of bacterial enumeration. As evident in Fig. 5D, MN-mediated vaccination of mice with p.a MNs resulted in MN dissolution and *in vivo* delivery of *P. aeruginosa* in murine ears. This resulted in a significant reduction in the bacterial load in the lungs of these mice 24 h post i.n challenge. Almost a two-log difference in lung CFUs was evident in the mice that received the p.a MNs, compared to their unvaccinated counterparts, those that received bMNs.

At 24 h post challenge, the number of CFUs in the spleens was overall very low in both groups (Fig. 5D), since the mice were sacrificed 24 h post challenge, possibly leaving insufficient time for bacterial dissemination to this organ. It is however, noteworthy that there was a marked reduction in the number of spleens infected with *P. aeruginosa* within the vaccinated group (three out of five spleens were found to be sterile), compared to the unvaccinated group, which received bMNs (one out of five spleens was found to be sterile). These results collectively demonstrate generation of a protective immune response *in vivo* following application of our dissolving polymeric MNs*,* resulting in enhanced bacterial clearance from the lungs and decreased likelihood of systemic dissemination of *P. aeruginosa*.

## 4 Discussion

The emergence of multidrug-resistant bacteria for which no vaccines exist, has resulted in high mortality rates with limited or no therapeutic interventions available (Jansen et al., 2018). Thus, there is a necessity for greater consideration for the role of preventative vaccines to combat these pathogens. Moreover, there is a requirement for alternative vaccine delivery mechanisms that circumvent the requirement for injection and cold chain storage. As such, utilising a simple, cost effective approach, *P. aeruginosa (*strain PA01) was used as a model bacteria, heat-inactivated and formulated into dissolving MNs. This study was conceived as a proof of concept study for investigating the feasibility of taking a highly pathogenic bacterium, incorporating it into dissolving MNs and successfully delivering the heat-inactivated bacterium *in vivo,* inducing protective immune responses with a view, in time of working towards more relevant bacteria of the developing world.

MNs were prepared using Gantrez®, previously shown to have been successfully used in the formulation of dissolving MNs for delivery of model protein antigens, importantly, without deterioration of the antigens upon release from the MNs (Zaric et al., 2013a, McCrudden et al., 2014, McCrudden et al., 2014b). MNs laden with a heat-inactivated bacterium and blank MNs were prepared and characterized using tests defined in previous works (Lutton et al., 2015). In summary, MNs were found to be mechanically sound and successfully inserted into the artificial membrane PF, retaining their needle structure upon removal. Moreover, OCT showed that the MNs were of sufficient strength to penetrate murine skin and the needles dissolved within the first 5 min of skin application. From a clinical viewpoint, rapid MN dissolution may be desirable for monitoring of possible anaphylactic reactions.

Bacterial load post challenge is related to survival status and, therefore, the enumeration of bacteria in vaccinated mice was investigated. MN i.d vaccination resulted in reduced bacterial load in the lungs and reduced bacterial dissemination to the spleens. These results are in line with those of Yang et al. who demonstrated reduced bacterial loads, inflammatory cytokine expression (IL-6) and inflammatory cell infiltration, following intramuscular (i.m) vaccination with POH and adjuvant, in a challenge pneumonia model (Yang et al., 2017). Yanyan and co-workers showed that vaccination with X-ray-irradiated inactivated vaccine also controls CFUs in the lung and bacteria spread to the main organs and blood of mice following challenge (Li et al., 2016).

The use of lyophilised heat-inactivated vaccines delivered *via* dissolving MNs is advantageous for i.d vaccination. Firstly, heat-inactivated vaccines are safe for administration to immunocompromised patients (Ljungman, 2012). Administration of attenuated vaccines to these patients could result in a heightened sensitivity response, and as such, inactivated cells represent an attractive alternative (Walker, 2005). Also, the thermostability of inactivated vaccines within MNs avoids the requirement of cold chain storage and its associated costs (Mistilis et al., 2017, Zaric et al., 2013). At present, inadequate fuel and transport, necessary to ensure continuous running of cold chain equipment, often leads to unreliable delivery of supplies and vaccine stock-outs within developing countries (Shen et al., 2014). Moreover, the use of MNs present several advantages over conventional needle and syringe delivery system. One of the advantages is the prospect for self-vaccination. This is an important advantage as effective vaccines are sometimes inaccessible in developing countries due to geographical accessibility and lack of trained personnel for correct administration. It is these countries that carry the greatest burden of infectious diseases and struggle with vaccine compliance (Oliwa and Marais, 2017). The Global Vaccine Action Plan (GVAP), approved by the WHO, identified the requirement for improved delivery approaches for vaccination and MNs may offer a possible solution (WHO | Global Vaccine Action Plan 2011–2020, 2012). Additionally, dissolving MNs avoid the generation of sharps waste (Arya and Prausnitz, 2016) and may overcome problems of needle phobia and subsequent vaccination non-compliance (Taddio et al., 2012).

The mechanism of vaccine delivery *via* dissolving MNs results in polymer deposition in the skin. Although repeated application of dissolving MNs will not be required for vaccine delivery, regulatory bodies will undoubtedly require assurance of polymer safety. Accordingly, an understanding of the long-term effects of polymer deposition will require further investigation to ensure they do not represent a danger. While dissolving MNs are typically fabricated from biocompatible polymers, they have not been used for i.d delivery. With the MNs utilised in this particular study, each MN deposits approximately 5.4 mg of polymer per array, which is the polymer content in the needles only. As highlighted such polymer deposition may result in effects such as erythema or hepatic/lymphatic accumulation and requires further investigation (Quinn et al., 2015). From a vaccination perspective, although prime-boost applications may be required, MNs will not require daily long-term application. Therefore, it is unlikely that such polymers will have significant long-term secondary/detrimental effects.

As this was a proof of concept investigation, studies were not carried out to elucidate the immunological mechanism by which p.a MNs confer protective immunity. This research was designed to develop and evaluate a pre-clinical MN delivery platform for i.d vaccination with heat-inactivated bacteria. Consequently, we describe a simple, cost effective approach to vaccination that could be applicable to a range of pathogens. Future studies are warranted to elucidate the mechanism of immune responses elicited post MN-mediated vaccination against *P. aeruginosa*. Such experiments should evaluate multiple cellular and humoral facets of the immune response and the cross-protective efficacy among different isolates of *P. aeruginosa.* Moreover, studies should also take into account the effects of polymer deposition and subsequent impact on immune response. In addition, prime-boost regimes and an investigation into long-term protection are warranted.

## 5 Conclusions

The work presented here reports successful formulation and characterisation of dissolving MNs laden with heat-inactivated *P. aeruginosa* for i.d vaccination. Notably, MNs were fabricated using pressure only, highlighting the simplicity and cost-effectiveness in the strategy employed. In addition, *in vivo* murine challenge studies confirmed delivery and the generation of an immune response, resulting in reduced bacterial load in the lungs and spleens of vaccinated mice. Together, these results suggest that dissolving MN-mediated i.d vaccination is a potentially viable delivery system. This technology could simplify vaccine administration and distribution in developing countries, thus improving vaccine coverage. Heat-inactivation of bacteria and its incorporation into MNs offers a novel, simplified approach to vaccination against a specific pathogen. This is the first reported study utilising a dissolving MN platform for successful delivery of a lyophilised heat-inactivated bacterium for vaccination against a pathogen for which no currently licensed vaccine exists.

This was an initial, but important study highlighting the potential of MNs for delivery of heat-inactivated vaccines. We anticipate that this dissolving MN system, due to its simplicity and ease of use, could potentially have a substantial impact on the prevention of infectious disease. Dissolving MNs offer the prospect of mass vaccination and the incorporation of heat-inactivated bacteria provides a simplified approach, which could easily be implemented in countries of the developing world. This was a proof of concept study and prior to this technology being applied to patients; several additional studies are warranted, including studies to elucidate the mechanisms of induced immune protection and patient usability/acceptability profiles. This technology clearly holds much potential and warrants further investigation.
